# Building barriers: The role of MYB genes in rice root adaptation

**DOI:** 10.1093/plcell/koae284

**Published:** 2024-10-22

**Authors:** Gwendolyn K Kirschner

**Affiliations:** Assistant Features Editor, The Plant Cell, American Society of Plant Biologists; The James Hutton Institute, Invergowrie, Dundee DD2 5DA, UK

As land plants evolved, they developed diffusion barriers in the apoplast to protect themselves from their surroundings and to separate different tissues. These barriers are created by strengthening the polysaccharide-based cell wall with extra hydrophobic materials. In roots, a hydrophobic layer in the primary cell wall of the endodermis, the Casparian strip (CS), seals the cell wall space between adjacent endodermal cells, playing a crucial role in preventing the undesirable inflow and backflow of nutrients between the soil and the root stele (Nawrath et al. 2013). In new work, **Xingxiang Chen and colleagues (Chen et al. 2024)** describe 4 Myb transcription factors that regulate suberization and nonlocalized lignification in the rice endodermis.

In rice roots, the CS forms ∼5 to 10 mm shootward of the root tip by lignification at the center of the anticlinal side in the endodermis cells (Wang et al. 2022). During rice root development, compensatory lignification has been observed at the edges of the endodermis cells facing the cortex and at the pericycle-facing side of the endodermis (lignin-based corner band). Subsequently, this compensatory and nonlocalized lignin fills the entire anticlinal cell wall of the endodermis cells (Fig.). At the same time, endodermal suberization coats all surfaces of the endodermal cells. This suberin is responsive to abiotic stimuli, including treatment with abscisic acid, cadmium, or salt (Chen et al. 2024).

**Figure.**
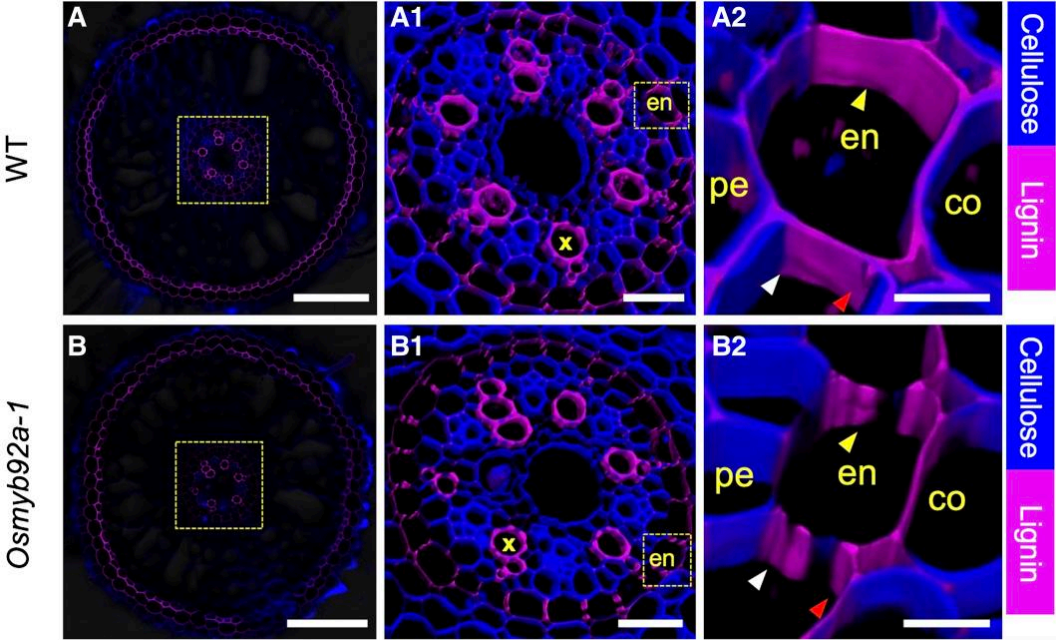
Suberization and nonlocalized lignification at the root endodermis in rice. **A)** In wild type (WT), nonlocalized lignin (magenta) fills the gap between the Casparian strip (yellow arrowhead) and the lignin-based corner band (white and red arrowhead) at the anticlinal side of the endodermal cells. **B)** In the *Osmyb92a* mutant, no such nonlocalized lignin deposition can be detected. A1, B1 show boxed area in A, B; A2, B2 show boxed area in A1, B1, respectively. Scale bars: 100 *μ*m (A-B), 20 *μ*m (A1-B1), and 5 *μ*m (A2-B2). Adapted from Chen et al. (2024), Figure 3.

In Arabidopsis, the MYB transcription factors AtMYB41, AtMYB53, AtMYB92, and AtMYB93 are key regulators of endodermal suberization under normal and stress conditions and also affect CS formation through a compensatory mechanism by production of nonlocalized lignin in the endodermis that is modulated by the CS integrity signaling pathway involving CS INTEGRITY FACTORs and SCHENGEN3 (Shukla et al. 2021). Unlike Arabidopsis, the molecular mechanisms in rice roots that control the interaction between endodermal lignification and suberization, as well as the relationship between suberization and environmental signals, are not well understood. Three OsMYB36 transcription factors have been shown to be involved CS formation, compensatory lignification, and suberization at the endodermis (Wang et al. 2022). Chen and colleagues now characterize 4 OsMYB36-dependent MYB transcription factors, the AtMYB41 homologs OsMYB39a, OsMYB41, OsMYB92a, and OsMYB92b, and analyze their role in the formation of diffusion barriers in the root (Chen et al. 2024).

Single and higher-order CRISPR-edited mutants of *OsMYB39a*, *OsMYB41*, *OsMYB92a*, and *OsMYB92b* caused defects in endodermal suberization and nonlocalized lignification deposition in primary and crown roots, under normal and stress conditions, affecting the plant's ability to manage stress conditions like abscisic acid, cadmium, and salt. However, CS functionality was not affected in these mutants. Overexpression of these MYBs within the endodermis enhanced lignification and suberization by early production in the endodermis, although this was not a functional apoplastic barrier. However, endodermal overexpression of *OsMYB92a* improved salinity tolerance by minimizing Na^+^ accumulation and the Na^+^ to K^+^ ratio in the shoots. OsMYB92a was identified as a master regulator, with its expression influencing various genes involved in suberization.

The study highlights the crucial role of OsMYB39a/41/92a/92b in controlling suberization and nonlocalized lignification at the rice endodermis. These proteins redundantly regulate multiple lignification and suberization-related genes and function downstream of OsMYB36a, OsMYB36b, OsMYB36c, OsCIF–OsSGN3, and stress-responsive signaling pathways. Enhancing endodermal suberin could be an effective strategy to improve the adaptation of rice to salinity stress.

## References

[koae284-B1] Chen X , Liu K, Luo T, Zhang B, Yu J, Ma D, Sun X, Zheng H, Xin B, Xia J. Four MYB transcription factors regulate suberization and non-localized lignification at the root endodermis in rice. Plant Cell. 2024:koae278. doi: 10.1093/plcell/koae278 39405464 PMC11663582

[koae284-B2] Nawrath C , Schreiber L, Franke RB, Geldner N, Reina-Pinto JJ, Kunst L. Apoplastic diffusion barriers in Arabidopsis. Arabidopsis Book. 2013:11:e0167. 10.1199/tab.0167 24465172 PMC3894908

[koae284-B3] Shukla V , Han JP, Cléard F, Lefebvre-Legendre L, Gully K, Flis P, Berhin A, Andersen TG, Salt DE, Nawrath C, et al Suberin plasticity to developmental and exogenous cues is regulated by a set of MYB transcription factors. Proc Natl Acad Sci U S A. 2021:118(39):e2101730118. 10.1073/pnas.2101730118 34551972 PMC8488582

[koae284-B4] Wang Z , Zhang B, Chen Z, Wu M, Chao D, Wei Q, Xin Y, Li L, Ming Z, Xia J. Three OsMYB36 members redundantly regulate Casparian strip formation at the root endodermis. Plant Cell. 2022:34(8):2948–2968. 10.1093/plcell/koac140 35543496 PMC9338812

